# Method for the Development of Accessible Mobile Serious Games for Children with Autism Spectrum Disorder

**DOI:** 10.3390/ijerph19073844

**Published:** 2022-03-24

**Authors:** Angel Jaramillo-Alcázar, José Arias, Israel Albornoz, Alex Alvarado, Sergio Luján-Mora

**Affiliations:** 1 Facultad de Ingeniería y Ciencias Aplicadas, Universidad de Las Américas, Quito 170125, Ecuador; jose.arias@udla.edu.ec (J.A.); israel.albornoz@udla.edu.ec (I.A.); alex.alvarado@udla.edu.ec (A.A.); 2 Department of Software and Computing Systems, University of Alicante, 03690 Alicante, Spain; sergio.lujan@ua.es

**Keywords:** accessibility, autism, disabilities, educational, impairments, learning, serious games, sustainability, ASD

## Abstract

Autism spectrum disorder (ASD) covers a range of neurodevelopmental disorders that begin in early childhood and affects developmental activities. This condition can negatively influence the gaining of knowledge, skills, and abilities, such as communication. Over time, different techniques and methods have been put into practice to teach and communicate with children with ASD. With the rapid advancement in the field of technology, specifically in smartphones, researchers have generated creative applications, such as mobile serious games, to help children with ASD. However, usability and accessibility have not been often taken into account in the development of this type of applications. For that reason, in this work we considered that both, usability and especially accessibility are a very important differentiators for the quality and efficiency of mobile serious games. Our approach has two important contributions, the incorporation of accessibility as a fundamental requirement in the development of a mobile serious game and the proposal of a method for the development of this type of applications for children with ASD, a method that can be used by other developers.

## 1. Introduction

One in every 160 children in the world suffers from an autism spectrum disorder (ASD) [1], which is characterized by deficits in social communication and social interaction, and by the incorporation of repetitive and ritualistic behaviors that are present in the early childhood and impair daily functioning [2]. Current treatment options are largely based on the treatment of comorbid psychiatric, neurological or medical conditions, as the core symptoms of autism are often refractory to current pharmacotherapeutic options [3]. Therefore, many attempts to develop novel treatments have been aimed at improving core features in the hope of possibly changing the natural course of the condition [4].

Currently, we live in a world in which Information and Communication Technologies (ICT) are present in a very direct way in our society and, for this reason, we consider important that education is not left out of this phenomenon. We have investigated the applications that can serve as a tool for children with ASD in order to help them respond to their educational needs in a different, attractive and innovative way. ICT focused at children with ASD aim to improve communication and language, allowing them to express their emotions and identify the emotions of others to improve their social interaction [5]. By allowing them to have a tool that helps them in their social insertion, the integration of children with ASD in their environment is more natural and, therefore, the development of their skills and their self-sufficiency increases [6]. Additionally, it is widely known that children with ASD symptoms have extraordinary abilities to work with digital technology [7]. Portable devices, touchscreens, apps and more are undoubtedly those factors that allow children with ASD to generate these digital skills [8]. Even today, there are many professionals with Asperger’s who work in IT departments, precisely because of the skills they demonstrate with technology [9].

In the last decade, several novel digital technologies have been released, especially mobile phones and smartphones; these technologies have transformed the way people around the world communicate and access information. Nowadays, there are more phones than people in the world [10]. With a continuing shortage of child psychiatrists both in the USA and around the world [11], the potential of using ICT to provide or improve services for children with ASD cannot be ignored. Like the rest of healthcare, digital revolution has impacted the ASD community, as mobile device-based software and smart device applications are constantly being developed and commercially available to children and their families [12].

In addition, the fact that with the coming of the COVID-19 pandemic, the physical relationship between children and doctor became less accessible, and with it the increasing use of an alternative to remotely access the health system. This alternative is called eHealth [13], and our project intends that it can be also used for this purpose, since the relationship between children and doctor is sought a lot, either remotely or in person.

However, few of the existing applications for ASD are offered in the literature to help inform their actual use in children. Smartphone apps are most often used to teach children with ASD a variety of skills [14,15,16]. In our research, we first researched and compiled a list of existing applications to see where there are gaps in topic coverage.

Currently, a mobile serious game aimed specifically at supporting communication within an environment for children with ASD is necessary, since communication is essential for any type of activity. The absence of communication and even the lack of inclusiveness in children with ASD results in negative consequences for members of society with this condition, such as low educational levels, depression, low income, and loss of confidence or self-esteem [17]. This fact is an incentive for the development of the project presented in this work. For this reason, a method for the development of accessible mobile serious games is presented.

## 2. Related Work

In recent decades, there has been a social and technological change [18], where ICT have given rise to a series of social practices of communication and information exchange to which schools have had to adapt. These good practices constitute what today is conceived as being literate [19]. ICT offer us many possibilities such as:

- Create more flexible elements for learning;
- Eliminate space and time barriers between teacher and student;
- Promote interactive scenarios and environments;
- Provide autonomous, collaborative and group learning;
- Access a large amount of information;
- Be a source of communication.

Likewise, using the new ICT, to do the same things as with traditional technologies, is a big mistake. New technologies allow us to do things completely different from those done with traditional technologies [20]; hence, a criterion for their incorporation cannot be exclusively the fact that they allow us to do things more quickly, automatically and reliably. With ICT, what we must try is to create new learning settings, not reproduce the traditional ones, and this necessarily happens for the transformation of the role of the teacher and the student [21]. The incorporation of new technologies into educational institutions will allow us new ways of generating, accessing and transmitting information and knowledge [22].

In recent years, there has been a lot of interest in incorporating ICT into the teaching and learning process of children with ASD [23,24,25]. Some research has pointed out that ICT offer these students a controlled environment, as they help structure and organize the interaction environment of the student with ASD by being configured as a highly predictable medium that offers understandable contingencies for the student [26,27]. All children are attracted to visual media, however, children with ASD can find it much more attractive due to their visual qualities in the information process [28].

With the evolution of ICT, new tools have emerged such as applications that are small computer programs that help the user in a specific task, be it professional, leisure, entertainment or any other nature. A study, based on an exhaustive research, presented the classification of apps that currently exist aimed at children with ASD [29]:

1. Applications for communication and language: These types of applications work as tools that help children develop their communication and speech skills;
2. Applications for emotions, interaction and social behavior: This variant, on the other hand, encourages inclusion in social groups as well as helps regulate the control of emotions;
3. Applications for games and leisure: These applications perform the entertainment function only, which is also a very important aspect that must be considered for children with ASD;
4. Applications for support tools: These applications work as support in teaching and coexistence towards children with ASD, for example, pictograms.

On the other hand, the search for similar applications is an important activity in which the different characteristics that applications aimed at people with ASD have in common can be identified. In this way, common themes can be addressed that will serve as a guide in the development of the application.

For the development of the project, several applications with relevant content were investigated as it would serve as a guide to develop our application. This search was carried out through academic databases as well as in mobile application stores for Android and iOS operating systems. These were the applications that served a lot as a guide to start the development of our project:

1. *TEApp* is a video game that supports the cognitive development and emotional intelligence of children with ASD through fun. Within the application, there are several exercises based on pictograms focused on the stimulation of different aspects of cognitive development [30].
2. *Dictapicto* is an app that allows the users to immediately pass a voice or written message to images. Designed to help children with ASD or those who use pictographic systems to improve their communication, its objective is to improve access to information and facilitate understanding of the environment regardless of whether the people around the children with ASD know these systems [31].
3. *PictoTEA* is an application specially designed to help children with ASD, Pervasive Developmental Disorder (PDD) or any other difficulty that affects social and communication skills in communication. On the other hand, it allows the users to customize the content in five stages with different degrees of difficulty in order to follow the person’s progress, adding pictograms, categories and even the option of putting together phrases [32].
4. *José Aprende* is an app supported by Fundación Orange. José Aprende is a collection of stories, visual stories adapted to pictograms and developed especially for children with ASD, also serving other learning difficulties and pre-reading children [33].
5. *TEAyudo a jugar* is an interactive application for mobile devices, a learning, intervention and social inclusion tool for children with ASD or other neurodevelopmental diversity. Through this app the user is provided with a complete tool to visually plant moments of games [34].
6. *Pictotraductor* is a project developed to facilitate communication with people who have difficulties of expression through oral language and who communicate more efficiently through images. Designed as a useful tool for parents and professionals, to be able to communicate, anywhere easily and efficiently [35].

The applications described served us a lot as a guide to start the development of our project. What we tried to do is to make an app a little easier to use and that, being focused on non-verbal children, has a verbal assistant that is the one that speaks for the child. In addition, accessibility was a important requirement in its development.

Unlike Dictapicto that performs the reverse process, that is, the user is the one who speaks and is translated into pictograms for the child, which is why we believe that both applications are complementary.

On the other hand, Pictotraductor helped us a lot with obtaining images and the Picture Exchange Communication System (PECS) that they tried to make the most attractive for the children based on what this web application showed us. However, there is also the PictoTEA app which is a somewhat more complete application, as it encompasses both principles. One feature of this app is the fact that it has levels in stages that increase as progress is made, which, unlike our project, does not seek to challenge so much the child but rather provides instant communication facilities in an ordinary day.

From the survey of existing smartphone applications targeting children with ASD, a set of patterns or guidelines can be drawn to be incorporated into a serious game for mobile devices that encourages children to interact in a social environment by answering simple questions of daily actions. These questions can include actions from personal hygiene, to actions that are somehow essential in everyday life. Topics such as food, outdoor activities, common requests, among others, can also be included in the application.

## 3. Method and Materials

The present study proposes the combination of two methods that allow the development of accessible mobile serious games to improve communication in children with ASD. Thus, as an application development scheme, it has been proposed to use the User-Centered Design method (UCD). UCD is a product design and development concept that focuses on user needs to enable an optimal user experience [36]. Additionally, through UCD, accessibility requirements can be incorporated. There are several reasons why we chose UCD as our design methodology. UCD works much better when the team involved in the design process is multidisciplinary, this means that having a team made up of people with different backgrounds and disciplines can lead to more creative design decisions. On the other hand, it is a methodology where the project team unites behind a common goal where the risk of project failure is mitigated as concepts and ideas are validated with users throughout the project. In addition, the project team narrows its focus to a smaller number of key features that are specific to the user’s needs [37].

The main reason for using UCD is that we believe it is the best option for the end user characteristics of the product. This is because “User-Centered Design is a broad term to describe design processes in which end-users influence how a design takes shape” [38]. In other words, the most relevant concept is that users are involved in one way or another. For example, using UCD, users are consulted about their needs and involved at specific times during the design process; typically during requirements gathering and usability testing. Still, there are extreme cases where users have a profound impact on design by engaging as partners with designers throughout the design process. Likewise, [38] identified three types of users: primary, secondary, and tertiary. Primary users are those people who actually use the artifact. Secondary users are those who will occasionally use the artifact or those who use it through an intermediary. Tertiary users are the people who will be affected by the use of the artifact or make decisions about its purchase.

On the other hand, the serious video game development method proposed in our previous work will be used [39]. This method condenses the elements that must be considered for the development of a serious video game and can be individualized depending on the disability it addresses, in this case children with ASD.

For the sake of comprehension, both methods are described below and will later be applied in a case study focused on children with ASD.

### 3.1. User-Centered Design Method

UCD is a product design and development concept that focuses on user needs to enable an optimal user experience. UCD method is the one we found ideal for the development of the app. This method is characterized by a multi-stage process that allows input and feedback, as the developers create the product [40,41,42]. This approach has been used successfully in different types of technological products, such as the development of websites [40], creating virtual environments [43] and interactive health technologies [44].

In addition, UCD offers several benefits [38], including:

- Products are more efficient, effective, and safe;
- Assists in managing users’ expectations and levels of satisfaction with the product;
- Users develop a sense of ownership for the product;
- Products require less redesign and integrate into the environment more quickly;
- The collaborative process generates more creative design solutions to problems.

**Figure 1:**
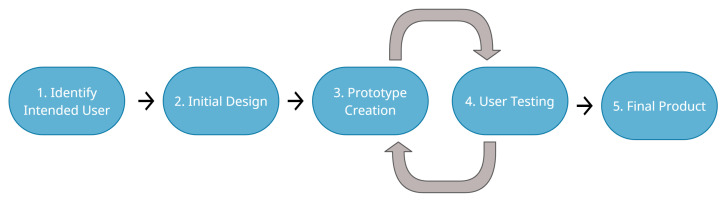
User-Centered Design Phases.

The user-centered design process is linear; however, the concept is multi-dimensional. Depending on the product and user population, the process should be adapted to the context of the purpose and type of software being developed [45]. This can be seen in Figure 1.

Below we describe the phases of the UCD, each of which will be applied in the case study of this work:

1. **Identify Intended User:** The first step in the user-centered design process is to identify the user of the software. Unified Modeling Language (UML) use cases are used to identify use situations with stakeholders. Additionally, in this step, the functional and non-functional requirements are collected or defined, included those related with the accessibility.
2. **Initial Design:** The initial design of a software should be created after getting feedback from all stakeholders related to the use of the application. In this way, many unique needs and desired capabilities of the application can be identified.
3. **Prototype Creation:** The prototype creation is the development of a prototype of the application.
4. **User Testing:** The user testing is the test or evaluation of the prototype creation. The prototype creation and the user testing are combined because it is an iterative integrative process in which for each improvement or version of the prototype, user tests are generated to validate their functionalities. In addition, accessibility is a parameter that must be considered and present in user tests.
5. **Final Product**: The final product is the result of the different adjustments made to each prototype generated from user tests. The final product will be considered with a version that can be disseminated and used by the end user.

### 3.2. Mobile Serious Game Design

**Figure 2:**
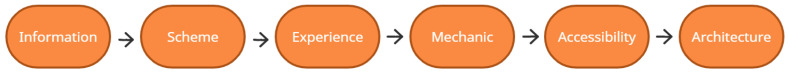
Serious Game Development Method [39].

As we said before, the development of the mobile serious game has been based on our method proposed in [39] where the relevant information was described to achieve a clear and efficient communication towards all those involved in the application: players, developers, publishers, among others. In Figure 2, the different elements proposed in the method are shown.

Each element collects the necessary information for the development of serious video games. *Information* collects the general information of the serious game such as the name, target age of the players, characters, among others. *Scheme* identifies the element or elements with which the player interacts to carry out the different challenges of the serious game. On the other hand, *Experience* allows to identify the type of experience that the serious game will offer the player, for example, the levels that it incorporates. *Mechanic* describes the information related to the playability of the serious game. *Accessibility* presents the accessibility guidelines that must be incorporated into serious gaming depending on the disability. Finally, *Architecture* describes the technological architecture that will support all the interaction of the elements of the serious game.

It is important to emphasize that the proposed method is flexible and allows selecting the most pertinent elements in the development of each type of application. This will depend on the type of serious game being designed and even the accessibility features required.

### 3.3. Quality Application Evaluation

Usability is an important attribute of mobile applications quality. For this reason, different methods have been developed in order to specify the level of usability and quality that mobile applications may have [46]. One of these methods is Mobile App Rating Scale (MARS). MARS is a tool designed for assessing the efficient use of mobile health applications. MARS was developed to determine the quality criteria of applications [47] and it has been used in several studies [48,49,50,51]. The MARS scale evaluates the quality of the application in four dimensions and 18 questions, with a grading similar to the Likert scale, that is, “1. Inadequate” to “5. Excellent”. The four dimensions and their corresponding items are described below:

- Engagement: entertainment, interest, personalization, interactivity and target group;
- Functionality: performance, ease of use, navigation and gesture design;
- Aesthetics: design, graphics and visual appeal;
- Information: Accuracy of the description of the application, objectives, quantity, quality of information, visual information and credibility.

## 4. Case Study

A case study is a research method used to answer how and why questions regarding a research topic [52]. There are many factors that affect the phenomenon of the case study of this research. These factors and their interactions are described below. Additionally, this case study is a valuable source of data given the complexity and purpose of our project. In this regard, it is important that the nature and focus of the case study are clear.

The general objectives of the case study in this research are:

- Describe the application of the selected methods in the design and implementation of a serious game that supports the communication of children with ASD;
- Identify key problems that the serious game might have in its use with children with ASDs;
- Analyze the serious game based on the possible results obtained from the evaluation by expert developers;
- Recommend a course of action to improve the serious game and its use for children with ASDs.

In addition, there are various techniques to engage users in the design and development of a product using UCD [38], such as the following:

- Background Interviews and questionnaires;
- Sequence of work interviews and questionnaires;
- Focus groups;
- On-site observation;
- Role Playing, walkthroughs, and simulations;
- Usability testing;
- Interviews and questionnaires.

All these techniques have been applied in the different stages of this case study. For example, interviews with therapists were generated to find out the requirements that an application aimed at children with ASD should have, with the aim of improving their communication.

In order to show feasibility and usefulness of the methods proposed in the previous section, this case study has been developed including all their stages. In each stage, the necessary elements have been included to obtain an application that can be used as a support for the communication of children with ASD.

SimpleTEA is an app that falls into two categories, the first is communication and language and the second is an app with a support tool character as it is designed for non-verbal children and, at the same time, seeks to be striking and innovative with the end user. It is also important to emphasize the fact that SimpleTEA is oriented to be a serious game, this implies that its goal is not only pure entertainment, actually what the app tries to promote is an education skill, and enhance a behavioral change [53,54,55], which, in this case, is communication.

Some studies [56,57,58] list guidelines for the design of software or serious games for children with ASD, established from the experience obtained from empirical studies, collaborations with therapeutic centers, interviews with parents, and user observations. SimpleTEA basically seeks to be developed in the same way, as it was developed through previous studies, and help from all parties involved in the life of a child with ASD. This includes parents, therapists and teachers, as well as the user experience while testing the app.

### 4.1. User-Centered Design Method

As indicated in the previous section, it is described below how each of the phases of the UCD method were applied for the development of this project.

**Figure 3:**
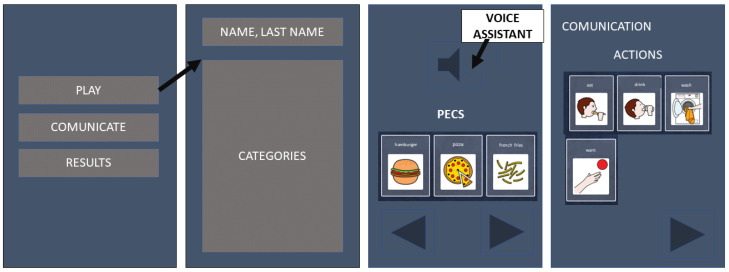
Prototype Design Tabs.

1. **Identify Intended User:** For this case, it is necessary to develop an application that is oriented to support in the communication of children who suffer from ASD and are also non-verbal. This app is a facilitating tool of requests or situations that the child may face throughout the day and, due to their condition, cannot be expressed in the proper way. For this reason, a type of voice assistant will be required to help the user in addition to requiring a childish and striking interface.
2. **Initial Design:** Initially, the design must be discussed and feedback from parents, therapists, teachers and even from people who have previously developed apps of this type, must be obtained. All these actors were able to communicate the concerns and difficulties that they were facing every day when dealing with children who have this disorder. It would have been helpful to be able to interact in person with the children in order to make a more personalized design within the application, however, due to COVID-19, this was not possible.
3. **Prototype Creation and User Testing:** The creation of the prototype as well as the user tests were treated as a single event, since they were developed with a deductive methodology based on trial and error. It was necessary for both therapists, parents and children to test the app and give their feedback on what is missing. For this reason, a type of design was temporarily created in the Microsoft PowerPoint tool demonstrating and consulting parents and therapists how the prototype seemed to them. This step intended to capture the idea that became apparent after the interviews we had with them. The design prototype can be seen in Figure 3, where the sketches of the main components, the home tab, as well as the PECS and the trivia and communication tabs are presented.
  After the conversation and feedback about it, it can be said that the design prototype was quite close to the final product as can be seen in the final version of the app. The design is essentially quite similar, despite the details and the aesthetics which are obviously more striking.
4. **Final Product**: The final result of this project is a tool of expression for a child with ASD. The drawback was that the project was designed with the objective of testing it in an institution that treats a large number of children with ASD. However, the impediment of the COVID-19 pandemic was the one that truncated the possibilities of testing several children and in a therapy environment as such. On the other hand, some attempts were made remotely that were successful, and despite not being as many as we wanted, it is a great start, which will surely bring great benefits for those who consider it appropriate to continue making applications of this nature.

### 4.2. Mobile Serious Game Design

For the design of a mobile serious game, it is important to identify how children with ASD understand the symbolic role of images and their learning through them. Initially, it has been concluded that many children with ASD have a different pictorial understanding compared to their typically developmental (TD) peers.

In [59], for example, it is indicated that children with non-verbal ASD and cognitive impairment at the time of using systems based on black and white images to communicate words, did not manage to extend the labels to the symbolized referents, different from children with TD [60]. However, in the study carried out in [61], it was found that a similar group of children with ASD extended labels to symbolized referents approximately twice as often in experiments with color images compared to experiments with images without color [62]. This clearly suggests that different types of images can affect children’s understanding.

On the other hand, in [63], information is presented where children with and without ASD learned names of unknown objects represented in photographs and subsequently classified them. TD children grouped items that matched shape, while children with ASD classified items by shape or color. These differences suggest that there could be an atypical way of learning words through pictures in children with ASD. In turn, this result leaves open the idea of whether the type of medium, digital or not, can affect the ability to understand symbols. Thus, although a child has difficulties assimilating verbal instructions, this is not necessarily true for visual instructions.

In addition, according to [64], a study was carried out on the color preferences that children with ASD highlight. In this case the, study revealed that the predominant color for both children with typical development is red while for children with ASD is blue, closely followed by red in the same way. That is why, for the design of the application, striking images will be used and interfaces will be created in which blue and red colors predominate.

#### 4.2.1. Information

Below are the elements that are part of the serious video game development method. Each element refers to our previously developed works [39]. The application of the method is detailed below:

- **Game title:** SimpleTEA. It is an application that is born from the union of the word “Simple” and TEA (for its acronym “Trastorno de Espectro Autista” in Spanish) which means autism spectrum disorder.
- **Intended game systems:** SimpleTEA allows the following systems:
  − Android OS from version 4.0.3 onwards;
  − iOS from version 8 onwards.
- **Systems requirements:** The minimum requirements that a cellphone requires to run SimpleTEA are:
  − 1 GB of RAM minimum;
  − 100 MB of storage space at least.
  Finally, it is important to mention that an Internet connection is required in order for the app to work successfully.
- **Target age of players:** SimpleTEA is recommended for 5 years old and older. The game is intended to be used for communication and also education.

#### 4.2.2. Scheme

**Figure 4:**
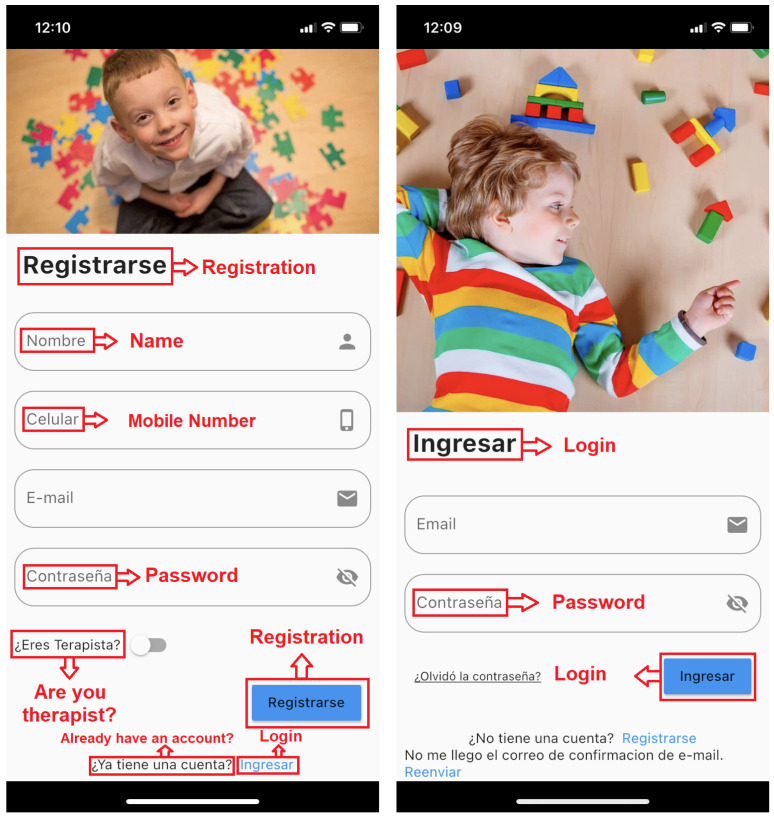
Registration and Login Screens.

**Figure 5:**
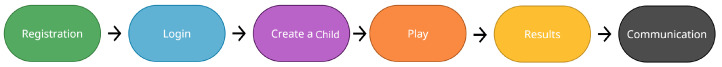
Serious Game Flow: (1) Registration, (2) Login, (3) Create a Child, (4) Play, (5) Results, (6) Communication.

**Figure 6:**
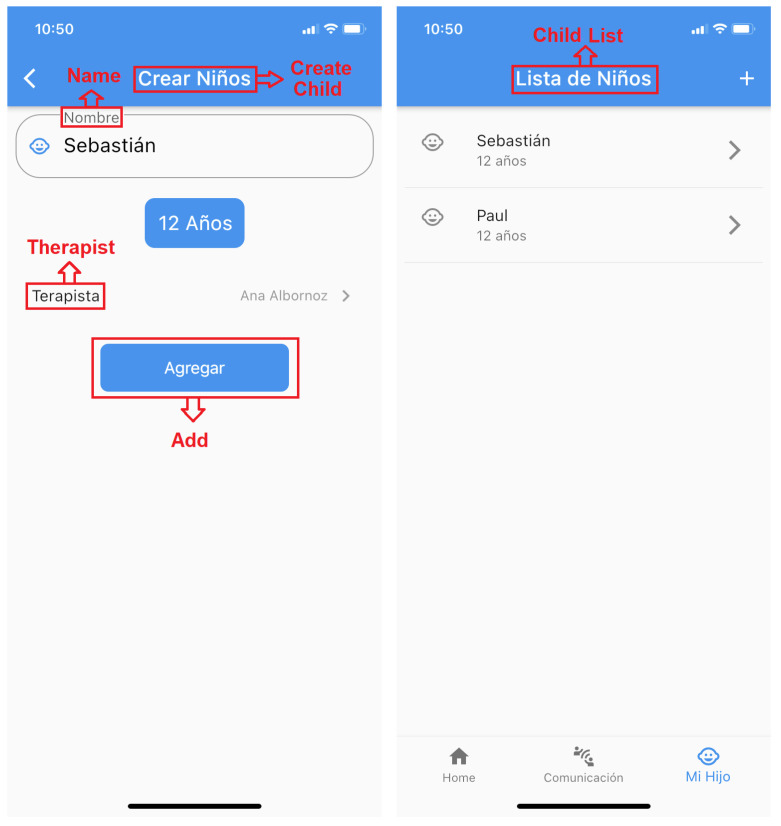
Child Creation and List of Children Screens.

- **Game story summary:** SimpleTEA is designed to operate only in a mobile environment. The game essentially has a registration and login module as shown in Figure 4. The current version is only available in Spanish, however, it is ready to be translated into other languages. Even in the figures, the translation of the texts into English has been included. Once registered, within the application, the user will have access to the game as such, as well as the communication sections and the list of children entered for the game.
- **Game Flow:**Figure 5 represents a diagram detailing the functioning of the game and order of how the app should be used in order to obtain better results:
  Next, we will describe the script that will follow according to what is shown on this diagram:
  **1**  **Registration:** This is the first thing the user should do in order to use the app, it is a common registration module in which names, mobile phone number, email account and a password will be required. There is also a therapist switch. In case that the user is a therapist, the switch should be turned on, and then, the administrator will validate if the user is a therapist or not. It is important to mention the fact that a validation email will also be sent in order to avoid fake accounts. The role of therapist is determined for the health professional who will be in charge of monitoring the child and generating the therapy sessions; likewise, the user will have access to the reports to verify the results of each of the sessions.
  **2**  **Login:** This element comes after the user signed up for the app and validated the email account, credentials must be entered and the application will open automatically.
  **3**  **Create a Child:** This section is required in order to start playing, there is no way to play without a child created. The information asked is the age and name of the child, and also the name of the therapist that is treating the child. This is evidenced in Figure 6.
  **4**  **Play:** This element is located in the *Home* tab and opens various categories available to the user. The child or the therapist can select any of them to later choose an appropriate level of difficulty.
  **5**  **Results:** Results will be shown in the child profile which is located in the *Create child* tab, the information displayed is intended to help the therapist to determine whether the child is learning or not.
  **6**  **Communication:** Once the child has learned or played several levels of the game, it is expected to use the words learned for communicating or asking things with all the people the child deals with every day.

#### 4.2.3. Experience

SimpleTEA seeks is to find a way to generate inclusiveness with children who suffer from ASD and are in turn non-verbal. The application intends that children and therapists have a new tool that performs the function of PECS, since this allows teachers to save on printed material or any other that is presented in a physical way, on the other hand, parents or guardians may also verify their child’s learning progress.

Once all this had been analyzed, the app began to be developed thinking about how applications are currently managed, creating an account to be able to use it. For this, the user needs a registration and login module like the one indicated in Figure 4. In turn, in the registration module, there is a switch for therapists in order to obtain that role. However, for security purposes and in order to avoid false accounts, a confirmation email was programmed. If this email is not verified, the account will not be operational.

Once the account is created, the child will be able to access the *Communication* tab. For this app, four categories considered the most important were generated. Additionally, there are a very wide number of words to start with. It should be noted that this application is a guide to continue developing other projects or applications of this nature [65].

**Figure 7:**
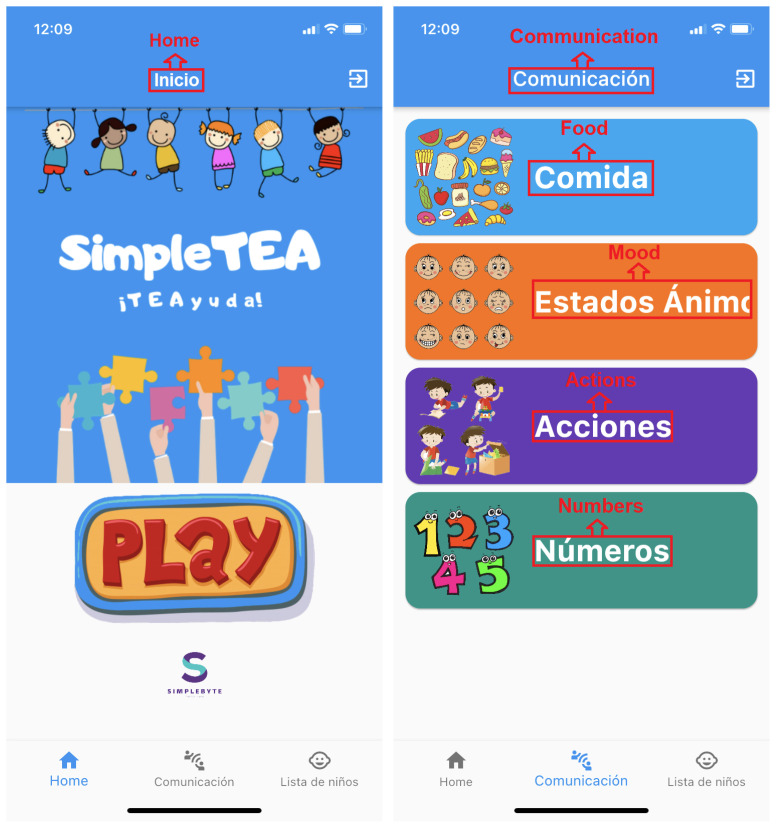
Home and Main Categories Screens.

In addition, in order to communicate, the child must first learn the words, this can be done from the *Home* tab where the app logo is displayed and, then, by pressing the *Play* button as shown in Figure 7 (left). On the other hand, in Figure 7 (right), the categories to be used as well as the *Home* tab, which lets the child access the trivia itself, can be evidenced.

**Figure 8:**
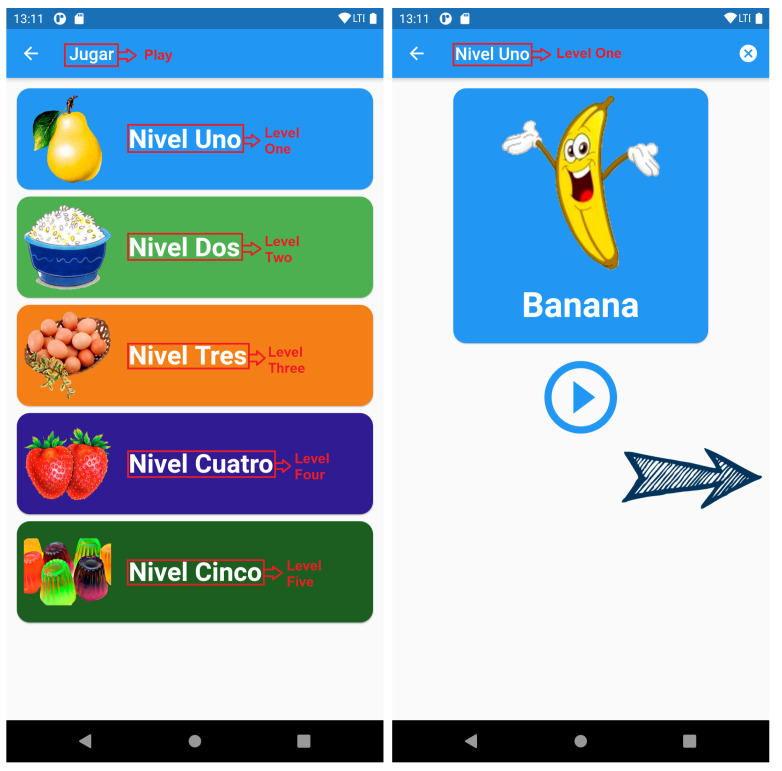
Food Category Levels and PECS and Voice Assistant Screens.

The game works as shown in Figure 8 (left). In each section, there will be levels that contain a variety of words according to the category, which does not necessarily increase the difficulty. This particularity seeks to minimize the number of words taught instead of introducing them all in a single section with the risk that confusion or anxiety is generated in the child.

As can also be seen in Figure 8 (right), the child is shown the image (PECS) and, at the same time, through a voice assistant, the child is instructed to relate the word to the image.

**Figure 9:**
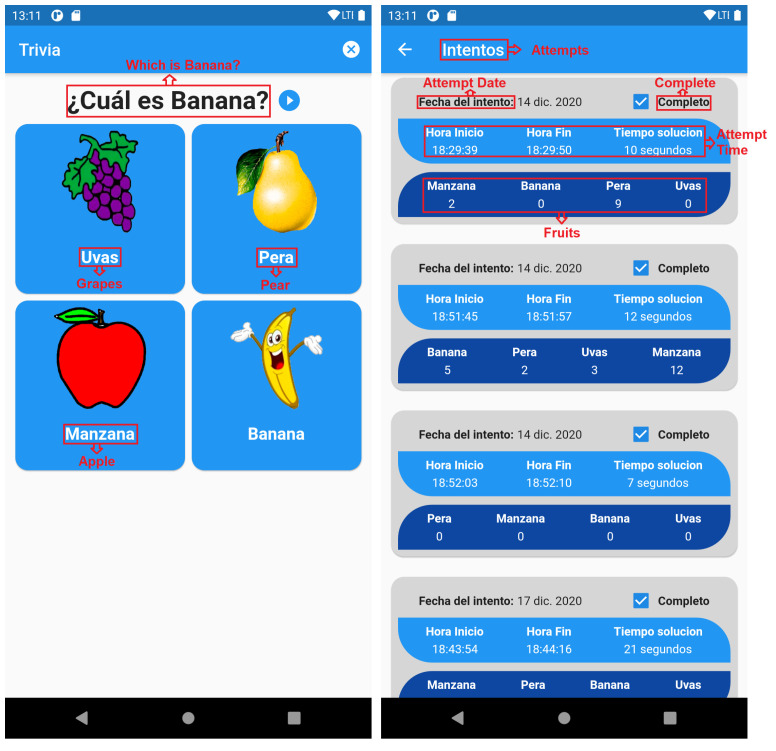
Question and Results Screens.

Later, as evidenced in Figure 9 (left), a small trivia is performed, asking if the child could relate the image to the word. This trivia in turn is repetitive, and is not sanctioned in case the child makes a mistake, simply ask again, until the child fully understands.

Once the trivia has been done, the results of the attempts in each of the trivia will be reflected in the section of the child’s profile as shown in Figure 9 (right). These results show how many times the question had to be repeated, the time that took the child to solve the trivia and the date in which the trivia was performed, and whether the child completed the test or not.

The results that can be verified by both the parent and therapist, help them to obtain feedback on the child’s learning, in what subjects the child should make a little more effort, which words are more difficult to learn and the time it is taking the child to learn about a certain subject. In this way, it is even possible to determine with more objectivity which subjects the child has a greater affinity with. All of this information can help those involved make smarter decisions about what to do next with the child to help their learning. In this way, parents and therapists have their own application available with a dashboard for consulting and analyzing all the data collected.

- **Objects:** The online serious game contains several decorative and interactive objects. Next, those that have some kind of interaction with the player are described:
  − **Pictograms:** These are the main elements of the game, the child must fully understand them in order to use them for communication in the future. The pictograms were obtained from real pictures used in real classes from professionals that use them on a daily basis.
  − **Voice assistant:** The voice assistant is a helper for the child, since they are not able to read. This kind of helper allows the child to understand what is being displayed on the screen.
- **Gameplay:** SimpleTEA provides the gameplay in the different platforms in which the online serious game can be executed: mobile devices (Android, iOS); on each platform, the controls are defined for each action required by the player, for example:
  − Mobility button: Forward (next page button);
  − Function buttons: Voice assistant button, correct picture button;
  − Exit button: To exit the trivia in case the child does not want to solve it anymore.
  In addition to the predefined options, a developer can add extra buttons on the screen to activate more features that the video game requires in the future, such as adding more pictograms or other special needs.

#### 4.2.4. Mechanic

In the case of people with motor disabilities, the interaction becomes more complex due to their condition. For this reason, if a child with ASD has a motor disability, it is recommended that an adult in charge helps the child to solve the game. Next, the elements related to the mechanics of SimpleTEA are explained:

- **Enemies:** There are no bosses or enemies within the online serious game, the app is intended to be educational and, above all, useful for the communication of a non-verbal child. The use of enemies had no sense in this game since it is a trivia-based game and also entertainment.
  However, there are several challenges that the child must face throughout the game, for example, learning most of the words through the pictograms in the shortest period of time possible.
- **MultiPlayer:** This online serious game is for a single player, but several can play it at the same time, or even several therapists can see their students results in the app at the same time as well. In SimpleTEA, teamwork was not considered as an objective because it is not what we intended to teach. The learning experience of it is actually private, personal and clearly individual, so that each user can go at their own pace. However, it is important to understand that a child with ASD needs help from all the parties involved in order to have a successful result and, in that way, the player truly receives and retains the information given.
- **Monetization:** The online serious game is free; however, it is not available yet to anyone who wants to download it, because it is intended to be customized for every institution that requires it, since every child is related to a personal therapist. This project does not seek an economic revenue for the moment, since it is a prototype that encourages the creation of more video games of this type following the idea already raised. However, in the future, if several institution consider it useful and want to customize the app, some charges may apply to help the sustainability of the project.

#### 4.2.5. Accessibility

The serious game accessibility considers some accessibility guidelines that were compiled in our previous work [66]. In that research, we analyzed the accessibility guidelines for serious video games aimed at people with cognitive disabilities.

For the compilation of requirements, the most relevant guidelines were analyzed, which are those described in the following items:

1. **Customizability:** The play experience should be highly adaptable to the child’s individual preferences and abilities. In SimpleTEA, this was difficult to accomplish considering that it is complicated to develop an application with the particularities of each user.
2. **Evolving tasks:** Increasing levels of motor or cognitive complexity must be incorporated into the game. In SimpleTEA, the categories are different from each other, motivating the user to memorize or relate the image to the word and thus generate the expected result.
3. **Single objective:** There must be a single explicit goal to achieve within a game session. In SimpleTEA, the objective is really simple and clear and the user only needs to learn the words through the PECS to be able to communicate in the future.
4. **Instructions:** Goals and tasks should be clear before playing and should not rely heavily on text or language. In SimpleTEA, the instructions are clear and intuitive and a voice assistant was incorporated so that the user is not stressed by the amount of text.
5. **Reward:** Offering a reward after a good performance increases the motivation, the commitment of the child and implicitly improves skills. Children with ASD prefer rewards that create fun, such as upbeat music and over-quantitative animations, rewards, for example, points or overtime. Penalties should be skipped when game performance is mediocre. In SimpleTEA, the reward is implicit in the fact of being able to communicate.
6. **Repeatability and Predictability:** Repeatability is important for mastering a skill and providing control of learning. Repeating the same tasks creates a certain predictability of the expected game objectives for the next game session. In SimpleTEA, the application is repetitive within the trivia and predictive since the structure is maintained throughout the application.
7. **Transitions:** Transition time between different game states should be minimized. Waiting can result in loss of concentration or attention. Transition screens should be kept simple to avoid fixations on repetitive elements. In SimpleTEA, the transition time is almost immediate both within the application and in the trivia.
8. **Minimalist Graphics and Clear Audio:** Graphics and audio should be aesthetically pleasing, but always functional. Irrelevant items can be distracting and can lead to loss of attention. Too many visual or auditory stimuli or colors could trigger anxiety. In SimpleTEA, the embedded images are as simple and minimalist as possible, as well as, the audio is clear and in line with the images.
9. **Serendipity:** Visual or audio effects can create wonders or surprise, resulting in more enjoyment of the game. However, sensory input must be predictable and consistent with certain tasks, for example, audio feedback for correct action with some fortuitous effects, for example, a new object that appears on the screen. It is important to find a balance between sensory stimuli to avoid loss of attention. In SimpleTEA, the voice assistant corrects the child’s actions in case of committing any mistake, not in a repressive but encouraging way.
10. **Dynamic stimuli:** Providing animations or music helps retain the child’s attention. A prolonged static vision, in on the other hand, can trigger unwanted behavior, such as stereotyped movements or motor stiffness, for example, looking at a static image on the screen. In SimpleTEA, music was implemented with pleasant sounds that manage to keep the child’s attention.

#### 4.2.6. Architecture

App development on multiple platforms has historically been difficult and complex [67]. However, we opted for developing the app in a tool called Flutter. Flutter is a tool that provides the developer instruments to create beautiful and professional-looking applications and with the ability to customize any aspect of the application [68].

**Figure 10:**
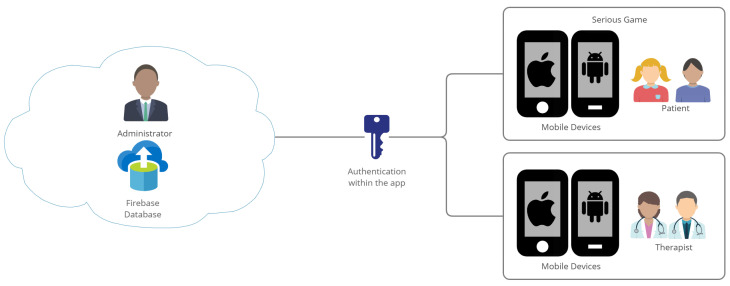
App Architecture design.

The architecture study for the application was carried out, in this case, the result was as follows, see also Figure 10.

Next, we explain the operation of the app based on the proposed architecture:

1. **Therapist role:** In this scenario, the therapist will have access to the functions of registration, login and create a profile of a child with ASD. In turn, the therapist has access to the data of the child’s father, mother, or guardian, and the results obtained in the different trivia that exist within the game.
2. **Patient role:** The patient’s scenario is different, since this will obviously be managed by the children parents or guardians, that is, the roles of child and parents are involved in one, because they will need an account to access the application; on the other hand, the child’s parents will be able to create a profile of their child, visualize their results and choose the therapist of their choice for their child to be treated.
3. **Administrator role:** The administrator is in charge of the management, control and maintenance of the database in the cloud, in this case, FireBase; however, he also has the obligation to approve a person to be a therapist since the requests will be reflected within the application with the role of admin.
4. **Aspects to consider:** People who take the role of therapist will be able to access their account, however, it will have the same children functionality as long as the administrator does not approve the request for the role. The child will need help from his/her parent or guardian to advance content within the application.

### 4.3. Quality Application Evaluation

For the quality evaluation of SimpleTEA, we use MARS [47]. MARS evaluates the quality of an application through 18 questions that make up four dimensions: engagement, functionality, aesthetics and information. The questions were answered by a group of five evaluators with experience in mobile application design with the aim of validating the usability of SimpleTEA, as can be seen in the Table 1.

**Figure 11:**
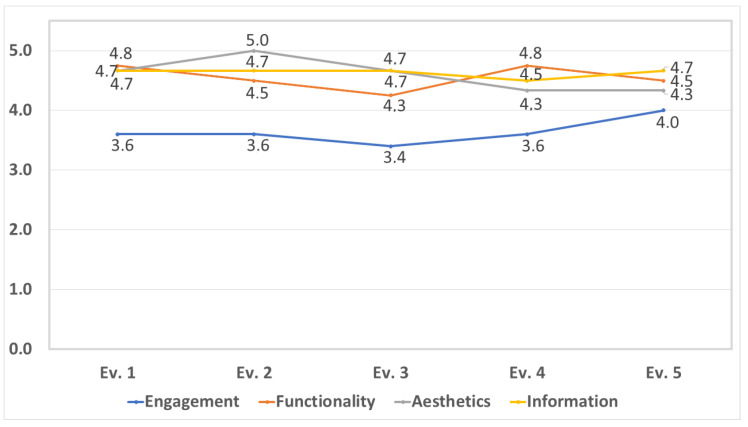
MARS Categories Evaluation.

Figure 11 has consolidated the evaluation carried out by grouping the results for each of the categories that MARS proposes. The evaluation allows us to identify that most of the criteria are met between values of 4 and 5, where the categories of functionality, aesthetics and information stand out. However, the category of engagement is the one that receives the lowest rating from all the evaluators with scores lower than 4. Without a doubt, it allows us to appreciate that the criteria that are part of this category need to be improved in the mobile application.

## 5. Discussion

In Table 2, we verify the compliance of the compiled guidelines in this study. In addition, we present an analysis about how the guidelines support the objective of the serious game.

Despite the fact that there are several applications in relation to ASD, it is relevant to emphasize how impactful SimpleTEA would be for children with this condition. This is important considering that video games have several benefits for children with ASD, for example, video games favor more social initiation behaviors than free play in children with ASD when they are played alone or in pairs [57]. For example, reducing stress and anxiety, which are among the most common psychiatric conditions in children and adolescents, with estimated rates of 16% in young people between the ages of 6 and 18 [69]. Severe stress or anxiety can disrupt daily life, school performance, and even relationships with peers and family. Some of the most common anxiety disorders in children with ASD are specific phobias, such as social phobia, obsessive-compulsive disorder and generalized anxiety disorder. It must be emphasized that these disorders occur in large numbers, due to the inability to communicate in children non-verbal.

Another impact that the project also seeks is to promote the reduction of the use of physical materials. Therapists do not usually have digitized tools, or in turn, do not know about effective applications for use in the classroom. They usually resort a lot to printed material and plastic objects, among others; this is not only inefficient, but also not very friendly to the planet. On the other hand, SimpleTEA seeks to digitize all kinds of materials, transforming it into a new initiative to treat children who have the disorder.

The advantage it offers is the versatility within the application, since the application achieves to explain the use of PECS, and to communicate with the people around them. This helps reduce stress and anxiety in children and is a great help at home. Unfortunately, SimpleTEA has not been tested in a sample of children as large as expected due to the COVID-19 pandemic. However, with the help of therapists and parents of children suffering from this disorder, we have identified that the application was useful for daily communication and daily use. This feedback has allowed us to identify another important point because many times, it is not taken with the seriousness required to generate inclusivity. The reason is because the development of applications of this nature shows an advance not only in a technological sense but also as a society. There is little development of solutions for disorders of this type and, by executing proposals such as those in this article, we are including those who have always been excluded.

At the same time, there are other aspects to be improved also within the app. The feedback from professionals for the next prototype or future development was that the application is capable of being parameterized to express phrases that allow the child to better communicate. This implies to incorporate a sequence of at least three images, separated by commas, so that the child can also generate their own phrases or have stored phrases that they use constantly for ease of use. The following option has also been considered: if the user has the possibility of accessing a physical database or a larger space in the cloud, new categories and images could be added according to the needs of each institution.

On the other hand, the results of having incorporated the MARS evaluation tool to verify the quality of SimpleTEA allowed obtaining relevant information for the study. First of all, it is evident that the features focused on the functionality, logic and navigation of the application are clear. Likewise, it has been validated that the graphic design, the color scheme and the visual appearance are adequate, considering the approach of the application. In addition, after the evaluation, it is evident that the objective of providing a tool for children with ASD is fulfilled and that the associated contents are relevant. Users can count on a quality application in terms of content and functionality that allows them to improve their communication regardless of ASD condition.

Finally, SimpleTEA is an application that aims to be private and personalized according to the needs of the institution where it is required to be implemented. For this reason, if at any point, commercialization is on the agenda, a new database must always be created for each specific educational center or therapy place, to create that bond between child and therapist.

## 6. Conclusions

This paper deals with the relation between a serious game and its accessible features for children with ASD. This initiative contributes to the improvement in education of people with disabilities. The use of accessible serious games supports the Article 24—Education of the United Nations (UN) Convention on the Rights of Persons with Disabilities [70] and the fourth Sustainable Development Goals of the UN—Quality Education [71], considering that it supports people with disabilities to have access to education on equal terms with people without disabilities.

This study aimed to design a method for the development of accessible mobile serious games for children with ASD in order to reduce stress and anxiety. In addition, this prototype evaluates the feasibility of designing a communication method between children with ASD and their parents, teachers and therapists. The results show that, although some guidelines are already available in many applications for children use, more research is needed on how to practically incorporate them into e-health applications, day after day. Thus, this generates more and more inclusiveness with children who suffer the disorder.

Currently, projects such as SimpleTEA promote the increase of accessible video games in the market. These works show that it is not so complicated to apply accessibility requirements in serious games for people with disabilities. In fact, the video game industry has begun to take into account the need to implement accessibility features [72], so that all players can fully enjoy the experience of video games without any restrictions.

The case study validates that the proposed method can be applied in initiatives that improve the lives of people with disabilities. The serious game development method used is very broad and can be replicated at any educational level and disability. This is one of the main motivators to continue generating similar projects.

In addition, the methods used in the development of SimpleTEA allowed to maintain a clear structure and direction throughout the execution of the project. On the one hand, UCD resolved the specific needs of the requirements defined for SimpleTEA in a coordinated manner, always thinking about the satisfaction of the end users, in this case children with ASD. Likewise, the method of developing serious games allowed us to capture the necessary elements, both in terms of gameplay and accessibility. In this way, it was possible to build a serious game that includes all the necessary elements to improve the communication of children with ASD.

In addition, for the design of the application proposed in this work, the support of families with children with ASD was necessary. In addition, this research shows the importance of periodic user tests that, due to the COVID-19 pandemic, could not be executed completely or successfully. This peculiarity became the key problem to be able to carry out a greater number of tests.

On the other hand, MARS allowed to verify the quality of SimpleTEA as a factor of great importance in this study. Features focused on application functionality, logic, and navigation scored high and are considered highly covered. Likewise, the graphic design, the color combination and the visual appearance were determined as adequate for the SimpleTEA approach. In addition, one of the main conclusions of this evaluation is that the serious game meets the objective of being a tool aimed at children with ASD, being supported by the content presented in the application.

Finally, one of the expected results and benefits of this project is the reduction of physical materials. This is considering that most therapists use printed materials to teach children with ASD. This effect occurs due to the lack of diffusion of digital tools such as SimpleTEA in the therapeutic field.

In the future, we intend to test this serious game with a bigger group of players in order to verify and measure its usability and effectiveness. An experimental group of children will be evaluated over a period of time, where the results reflected in the improvement of their cognitive abilities will be evaluated. Likewise, it will be considered a priority to evaluate the level of learning of users with this type of video games to verify its impact on people with disabilities, specifically in this case with ASD. In addition, we intend to implement new accessibility features, games for different treatments, and compatibility for more devices or software, such as PC, MAC and iOS, in order to have a multiplatform serious game. In addition, the necessary resources will be analyzed to implement an infrastructure that allows expanding the number of categories and images according to the needs of therapists and institutions. Finally, the ability to include multiple players or to generate a collaborative game will be a feature incorporated in future versions.

## Figures and Tables

**Table 1:** MARS Evaluation of SimpleTEA by five evaluators.

	Variables	Ev. 1	Ev. 2	Ev. 3	Ev. 4	Ev 5.
**Engagement**	E1. Entertainment	4	3	3	4	4
	E2. Interest	4	3	3	4	4
	E3. Customization	3	5	3	3	4
	E4. Interactivity	3	2	3	3	3
	E5. Target Group	4	5	5	4	5
**Functionality**	F6. Performance	5	4	4	5	5
	F7. Ease to use	5	5	4	4	5
	F8. Navigation	4	4	5	5	4
	F9. Gestural Design	5	5	4	5	4
**Aesthetics**	A10. Layout	4	5	5	4	5
	A11. Graphics	5	5	4	5	4
	A12. Visual Appeal	5	5	5	4	4
**Information**	I13. Accuracy of app description	5	5	5	5	5
	I14. Goals	4	4	5	4	5
	I15. Quality of information	5	4	4	5	4
	I16. Quantity of information	4	5	5	4	4
	I17. Visual Information	5	5	4	4	5
	I18. Credibility	5	5	5	5	5

**Table 2:** Guideline Verification.

Guideline	Compliance	Analysis
Customizability	✓	The game can be very adaptable to each individual, or to each child; however it is complex to create an application that is highly customizable.
Evolving Tasks	✗	Although the application as such does not increase the difficulty levels of the activities itself, the categories involved are different from each other, motivating the child to be able to memorize or relate the image to the word and thus generate the expected result and the user can communicate with the people around, such as parents, therapists, and teachers.
Single Objective	✓	The application fully complies with this guideline, because the objective of it is really simple and clear, the child only needs to recognize that he must learn the words through the PECS to be able to communicate in the future.
Instructions	✓	In the same way as the previous point, the instructions are also clear and intuitive; in addition, there is a voice assistant, so the child will not be stressed by the amount of text.
Reward	✗	This guideline was not solidified throughout development because a type of reward could not be correctly adapted for the child, despite the fact that the reward is implicit in the fact of being able to communicate, within the application there is no such reward.
Repeatability and Predictability	✓	The application is repetitive and predictive, as all the levels and categories are designed in a standard way, the application is essentially repetitive within the trivia and predictive, since the structure is maintained throughout the app.
Transitions	✓	The transition time within the app when teaching the words to the child is almost immediate, in the same way as the trivia, it is automatic every time the next question is answered, it is instantly displayed.
Minimalist Graphics and Clear Audio	✓	Given that the application itself focuses a lot on the child successfully memorizing the word, the images chosen for the development of the application are as simple and minimalist as possible, in the same way, the audio is clear and aligns with the images.
Serendipity	✗	At the end of each trivia, an image is displayed that basically indicates that the level was fully met; however, there is not so much intrigue and surprise within the application, on the other hand, the voice assistant does correct the child’s actions in case of committing any mistake, obviously not in a repressive but encouraging way.
Dynamic Stimuli	✗	In this section, within the design, music with pleasant sounds was implemented that manages to maintain the child’s attention, on the other hand, the images are static and lack animation, this pattern can however be complemented with the help of the therapist or family member in charge of the child stimulating his attention.

## Data Availability

Not applicable.

## Abbreviations

The following abbreviations are used in this manuscript:

ASD	Autism Spectrum Disorder
ICT	Information and communications technology
MARS	Mobile App Rating Scale
PDD	Pervasive Developmental Disorder
PECS	Picture Exchange Communication System
TD	Typical Development
UCD	User-Centered Design
UML	Unified Modeling Language
UN	United Nations
